# Increased expression of Tbet in CD4^+^ T cells from clinically isolated syndrome patients at high risk of conversion to clinically definite MS

**DOI:** 10.1186/s40064-016-2510-0

**Published:** 2016-06-18

**Authors:** Sharee A. Basdeo, Siobhan Kelly, Karen O’Connell, Niall Tubridy, Christopher McGuigan, Jean M. Fletcher

**Affiliations:** Schools of Biochemistry and Immunology and Medicine, Trinity Biomedical Science Institute, Trinity College Dublin, 152-160 Pearse Street, Dublin 2, Ireland; Department of Neurology, St. Vincent’s University Hospital, Elm Park, Dublin 4, Ireland; School of Medicine and Medical Science, University College Dublin, Dublin 2, Ireland

**Keywords:** Clinically isolated syndrome, Multiple sclerosis, TOB1, T cells, Tbet

## Abstract

**Background:**

The ability to identify clinically isolated syndrome (CIS) patients at high risk of progression to clinically definite multiple sclerosis (CDMS) would be clinically beneficial. The initiation of T cell mediated autoimmune diseases such as multiple sclerosis (MS) requires the initial inappropriate activation and differentiation of auto-reactive CD4^+^ T cells. The quiescence of naive T cells is actively maintained by molecules such as TOB1, which control the threshold of activation. Upon activation, CD4^+^ T cells can differentiate into various subsets depending on the milieu present. Th1 and Th17 cells are strongly implicated in MS, while regulatory T (Treg) cells constrain autoimmune inflammation and prevent autoimmunity.

**Findings:**

We therefore investigated the expression of TOB1, CD44 and Treg, Th1 and Th17 transcription factors in relation to CIS progression. The expression of *TOB1*, *CD44*, *FOXP3*, *TBX21* and *RORC* genes were measured in CD4^+^ T cells from 10 healthy controls, 20 CIS patients within 3 months of initial clinical presentation and 10 relapsing remitting MS patients sampled within 2 months of relapse. CIS patients were subsequently grouped into those who converted to CDMS within 1 year and those who remained CIS. No differences in the expression of *TOB1*, *CD44*, *FOXP3* and *RORC* were observed. There was a significant increase in the expression of the Th1 transcription factor Tbet, encoded by *TBX21*, in CIS patients that converted within 1 year compared with those who did not.

**Conclusion:**

This pilot data suggests a role for Th1 cells in CIS progression and warrants further evaluation in a larger cohort.

## Background

Clinically isolated syndrome (CIS) refers to the first neurological episode caused by central nervous system (CNS) demyelination which results in clinical presentation of a patient. Between 30 and 75 % of CIS patients will progress to clinically definite MS (CDMS) (Miller et al. 2005; Nilsson et al. 2005; Optic Neuritis Study Group 2008), characterised by CNS lesions that are disseminated throughout the brain and spinal cord, and accumulate progressively with time. It would be of great benefit to have a biomarker to identify CIS patients with high risk of conversion to CDMS as this would facilitate early treatment to ultimately delay or prevent clinical conversion to CDMS. No such laboratory biomarker currently exists, other than magnetic resonance imaging.

CD4^+^ T cells play a central role in the pathogenesis of MS, and the initial activation of naive auto-reactive CD4^+^ T cells is thought to be a key step in the initiation of disease. Both the Th1 and Th17 subsets play a key role in MS and its mouse models; Th1 cells produce the cytokine IFN-γ while Th17 cells secrete IL-17 (Fletcher et al. 2010).

There are a number of peripheral tolerance mechanisms that help to prevent the inappropriate activation of naive T cells that are specific for self-antigens. One such tolerance mechanism is the requirement for co-stimulation, where the ligation of CD28 on the naive T cell with B7.1 and B7.2 provides the second signal for T cell activation. In the absence of an infection, co-stimulatory molecules such as B7.1 and B7.2 are not usually expressed by antigen presenting cells and this helps to prevent the activation of self-antigen specific T cells. Naive T cells are actively maintained in a quiescent state by anti-proliferative mechanisms. TOB1 (transducer of ERBB2) is constitutively expressed in naive T cells and is down regulated upon full T cell activation (Tzachanis et al. 2001). Steady state expression of TOB1 controls the threshold of activation for CD4 T cells, since in vitro silencing of TOB1 abrogates the requirement for co-stimulation and over expression of TOB1 inhibited T cell proliferation (Tzachanis et al. 2001). In addition, deletion of TOB1 in mice resulted in severe CNS autoimmunity (Schulze-Topphoff et al. 2013) and reduced TOB1 expression has been associated with progression of MS (Corvol et al. 2008). Since TOB1 regulates the threshold of activation of naive T cells; acting like a brake to ensure cells do not become activated in response to inappropriate stimulation, reduced TOB1 expression in T cells is implicated in the development and progression of autoimmunity (Baranzini 2014). CD44 is a glycoprotein expressed at high levels on effector-memory type cells, and is involved in migration, adhesion and cell–cell interaction (Shimizu et al. 1989). CD44 ligands include osteopontin, hyaluronic acid and collagen (Naor et al. 1997; Weber et al. 1996). CD44 expression was previously shown in a microarray study to be increased, whereas TOB1 expression was decreased, in T cells from CIS patients that showed rapid progression to CDMS (Corvol et al. 2008).

Therefore in this study we retrospectively examined the expression of TOB1 and CD44 in a small pilot cohort of CIS patients with different conversion rates in order to determine if these markers are sufficient to predict CIS patients at high risk of rapid disease progression and therefore warrant a larger follow-up study. We also measured the gene expression of the transcription factors *FOXP3*, *TBX21* and *RORC* which are the master regulators of Treg, Th1 and Th17 cells respectively.

## Methods

### Patient information

Patients were identified from an MS patient database in St. Vincent’s University Hospital and invited to participate in the study. From the database we generated lists of all patients meeting the inclusion criteria described below for each arm of the study and then randomly selected 10 patients from each group based on their anonymised case record number to approach regarding participation. Informed consent was obtained for all participants. Baseline demographics and diagnosis history was recorded. The study population was composed of 4 groups:

1. Patients with CIS and no new clinical or radiological disease activity seen after 12 months (n = 10)
2. Patients with CIS who converted to CDMS within 12 months (n = 10), identified by clinical relapse.
3. Patients with established RRMS who were treatment naive and had converted from CIS within 12 months (n = 10)
4. Age and sex matched healthy controls (n = 10)

All patients were sampled within 3 months of their initial episode (if CIS) or within 2 months of a clinically confirmed relapse if RRMS. All patients were treatment naive at the time of sampling. Ethical approval for this study was obtained from the St. Vincent’s University Hospital Ethics Committee.

### Cell isolation

PBMC were isolated by density gradient centrifugation from patients and age and sex matched healthy donors, with informed consent. PBMC were cryopreserved for later analysis. CD4^+^ Th cells were magnetically sorted by negative selection (Miltenyi Biotech). CD45RO^−^ (naive) T cells were then isolated from total CD4^+^ T cells by magnetic depletion of CD45RO^+^ memory T cells (Miltenyi Biotech). The purity of sorted cells was routinely >97 %.

### RT-PCR

Expression of TOB1, CD44, Tbet and RORC in total and naive CD4^+^ T cells was examined by RT-PCR. Total RNA was extracted from sorted total and naïve CD4^+^ T cells using a miRNeasy extraction kit (Qiagen, Hilden, Germany) and treated with DNase I to eliminate genomic DNA contamination. RNA was quantified using the NanoDrop (Thermo Scientific). Reverse transcription to form cDNA was performed using a High Capacity cDNA reverse transcription kit (Applied Biosystems). Gene expression was examined with the 7900HT Fast Real-Time PCR system (Applied Biosystems) using pre-designed Taqman gene expression assays with AmpErase-free TaqMan Universal PCR master mix (Applied Biosystems). The appropriate house-keeping gene was selected from a panel by geNorm analysis; GAPDH obtained an M-value of less than 1.5 and was, therefore, considered stable enough to act as the normalising internal control in this study. The following primers attached to a FAM dye were used; *TOB1* (Hs03986111_s1), *CD44* (Hs01075862_m1; exon boundary 1–2 all isoforms), *FOXP3* (Hs01085834_m1), *RORC* (Hs01076122_m1) and *TBX21* (Hs00203436_m1) (all Applied Biosystems). The reference gene primer, *GAPDH* (Hs99999905_m1) was conjugated to the VIC dye. The data were normalised to *GAPDH* expression and analysed using SDS 2.3 RQ Manager (Applied Biosystems) software.

The *GAPDH* crossing threshold (CT) value for individual samples was subtracted from the target gene CT value. If the *GAPDH* endogenous control failed to cross the threshold after 33 cycles, the sample was excluded. The healthy control samples were averaged to use as a calibrator and patient data were expressed relative to this. Patients with values >1 indicated increased gene expression and those with results <1 indicated patients with decreased gene expression relative to healthy controls.

Statistical analyses were performed using Prism 5 software; groups (including healthy controls) were compared by two-way ANOVA with a Tukey post-test. p values <0.05 were considered significant and denoted in the figures with an asterisk.

## Results

### Distribution of patient demographics and onset of symptoms in patient sub-groups

Baseline characteristics, as demonstrated in Table 1 are consistent with typical disease characteristics with the exception of an over representation of transverse myelitis patients in the non-converting CIS population, however the numbers of patients in each group are small and we do not think this represents any clinically meaningful variation from the early conversion group.

**Table 1:** Baseline demographics and symptoms onset in each of the patient sub-groups

	CIS—not converted (n = 10)	CIS—converted (n = 10)	RRMS (n = 10)	HC (n = 10)
Mean age at onset (SD)	31.6 (6.4)	27.4 (5.2)	29.7 (6.9)	30.8 (4.6)
Men/women	5/5	3/7	4/6	3/7
Symptom onset				
Optic neuritis (%)	2 (20)	6 (55)	6 (60)	N.A.
Brainstem (%)	3 (30)	3 (27)	2 (20)	
Spinal cord (%)	5 (50)	2 (18)	2 (20)	

*CIS* clinically isolated syndrome, *RRMS* relapsing remitting MS, *SD* standard deviation

### The expression of TOB1 and CD44 mRNA in total CD4^+^ T cells and naive CD4^+^ T cells from CIS patients

**Fig. 1:**
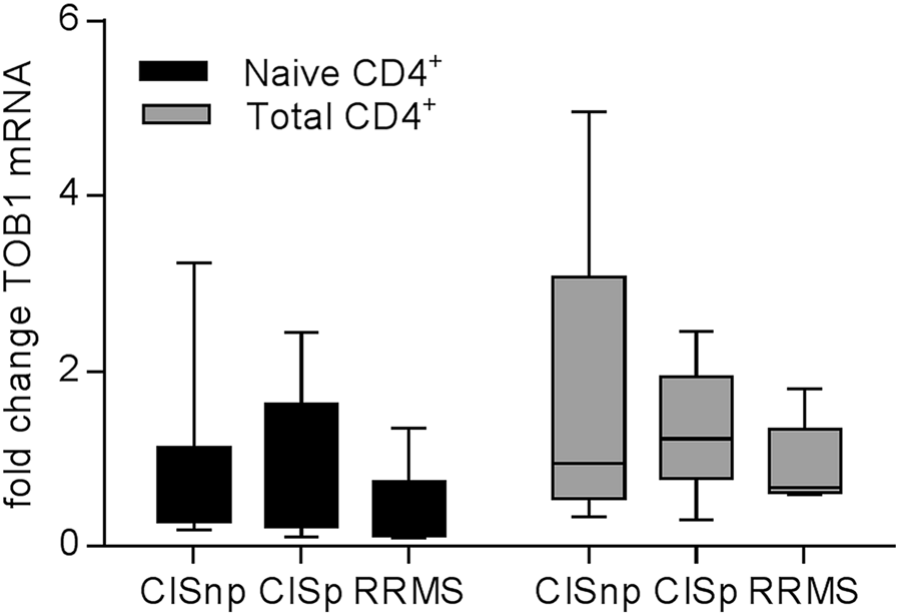
The expression of TOB1 mRNA in CD4^+^ T cells from CIS patients. The expression of TOB1 was assessed by RT-PCR in naïve (*black*) and total (*grey*) CD4^+^ T cells from healthy controls, CIS patients within 3 months of their initial episode and RRMS patients within 2 months of a clinically confirmed relapse who had previously converted to CDMS within 1 year. CIS patients were subsequently divided into those who converted to CDMS within 1 year (CISc) and those that did not convert (CISnc). All values are expressed relative to those of healthy controls averaged and set to 1. Statistical differences between groups were determined by two-way ANOVA with a Tukey post-test, however, results were not significantly different

Since TOB1 is thought to control the threshold of activation of CD4^+^ T cells, we sought to determine whether expression of TOB1 was altered in CIS patients that subsequently converted to CDMS within 1 year of initial presentation. Total CD4^+^ T cells and naive CD4^+^ T cells were isolated from cryopreserved PBMC samples; RNA was isolated reverse-transcribed into cDNA. The expression of TOB1 and CD44 was determined by RT-PCR relative to the housekeeping gene GAPDH. CIS patients were divided into those that subsequently converted to CDMS within 1 year of their CIS and those that did not convert within 1 year. For comparison, a group of established RRMS patients who had previously converted to RRMS within 1 year were included. The data were normalised relative to a group of age and sex matched healthy controls. No significant differences in relative expression of TOB1 were observed between CIS patients that converted within 1 year, those that did not convert and RRMS patients (Fig. 1). However, there was a slight trend towards reduced expression of TOB1 in the RRMS group.

**Fig. 2:**
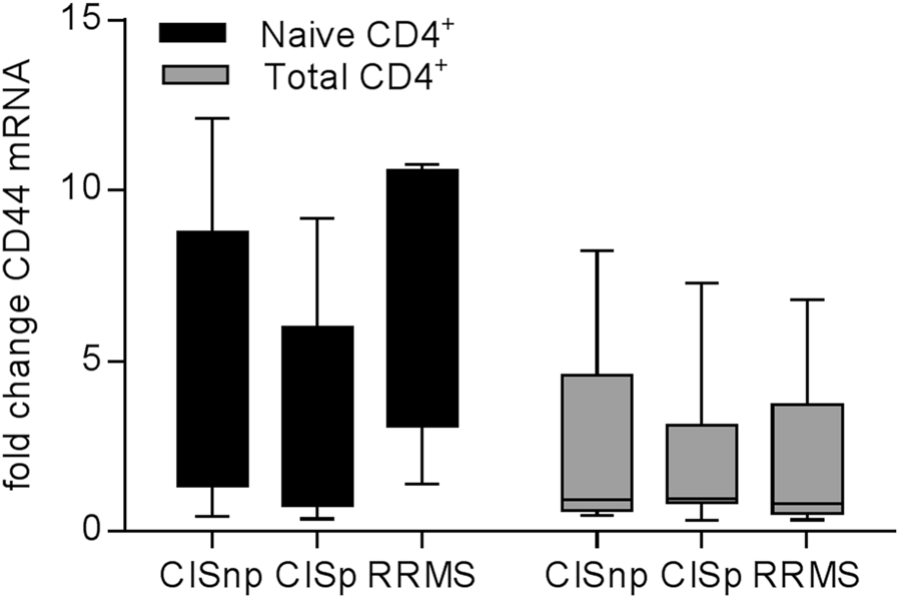
The expression of CD44 mRNA in total CD4^+^ T cells and naive CD4^+^ T cells from CIS patients. The expression of CD44 was assessed by RT-PCR in naïve (*black*) and total (*grey*) CD4^+^ T cells from healthy controls, CIS patients within 3 months of their initial episode and RRMS patients within 2 months of a clinically confirmed relapse who had previously converted to CDMS within 1 year. CIS patients were subsequently divided into those who converted to CDMS within 1 year (CISc) and those that did not convert (CISnc). All values are expressed relative to those of healthy controls averaged and set to 1. Statistical differences between groups were determined by two-way ANOVA with a Tukey post-test, however, results were not significantly different

Similarly, no significant differences in relative expression of CD44 were observed between CIS patients that converted within 1 year, those that did not convert and RRMS patients (Fig. 2). Interestingly however, the expression of CD44 was significantly increased overall in CD4^+^ T cells from patients in all three groups compared to controls (i.e. all relatively higher than 1).

### The expression of transcription factors FOXP3, RORC and Tbet in CD4^+^ T cells from CIS patients

**Fig. 3:**
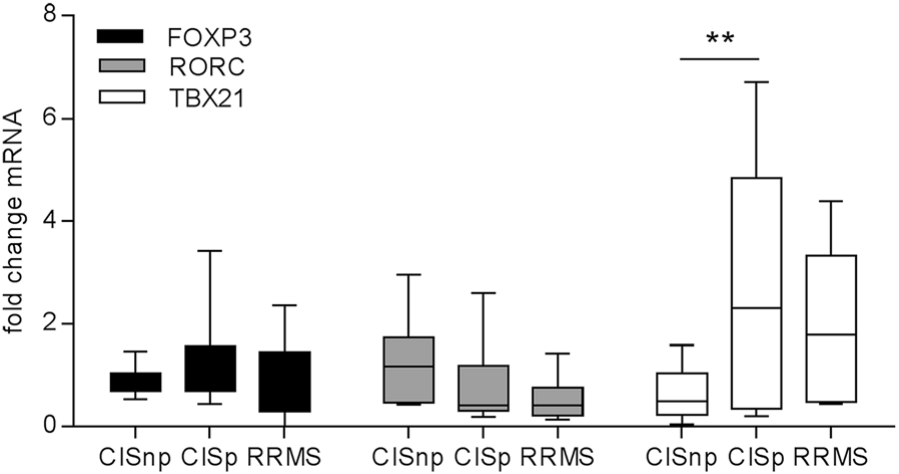
The expression of FOXP3, RORC and Tbet in CD4^+^ T cells from CIS patients. The expression of FOXP3 (*black*), RORC (*grey*) and Tbet (*white*) was assessed by RT-PCR in total CD4^+^ T cells from healthy controls, CIS patients within 3 months of their initial episode and RRMS patients within 2 months of a clinically confirmed relapse who had previously converted to CDMS within 1 year. CIS patients were subsequently divided into those who converted to CDMS within 1 year (CISc) and those that did not convert (CISnc). All values are expressed relative to those of healthy controls averaged and set to 1. Statistical differences between groups were determined by two-way ANOVA with a Tukey post-test; *p < 0.05

Since both Th1 and Th17 subsets of CD4^+^ T helper cells are implicated in the initiation and pathogenesis of MS, we also investigated the expression of the transcription factors Tbet and ROR-γt (encoded by the RORC gene) that act as master regulators for Th1 and Th17 cells, respectively. In addition we examined the expression of FOXP3, the key transcription factor of Treg cells, which help to constrain autoreactive T cells and prevent autoimmunity. Total CD4^+^ T cells and naive CD4^+^ T cells were isolated from cryopreserved PBMC samples, RNA was isolated and cDNA transcribed. The expression of Tbet, RORC and FOXP3 mRNA was determined by RT-PCR relative to the housekeeping gene GAPDH. CIS patients were divided into those that had subsequently converted to CDMS within 1 year of their CIS and those that did not convert within 1 year. For comparison, a group of RRMS patients who had previously converted to RRMS within 1 year were included. The data were normalised relative to a group of age and sex matched healthy controls. No significant differences in relative expression of RORC or FOXP3 were observed between any of the groups (Fig. 3). However, the expression of the Th1 transcription factor Tbet was significantly increased in CD4^+^ T cells from CIS patients that had converted (p < 0.001) relative to CIS patients that had not converted (Fig. 3). There was also a non-significant trend towards increased Tbet expression in the RRMS group relative to healthy controls and CISnc (Fig. 3).

## Discussion

The ability to identify CIS patients at high risk of conversion to CDMS would facilitate early treatment with disease modifying therapies that could prevent their progression and potentially improve patient outcomes. Since the activation and differentiation of pathogenic auto-reactive CD4^+^ T cells is considered to be a key step in the initiation of MS, we investigated the expression of key molecules involved in these processes. Interestingly we found that the expression of Tbet, the transcription factor that regulates the function of Th1 cells, was significantly increased in CIS patients who subsequently converted to CDMS within 1 year compared with those who had not converted. The expression of Tbet is induced during the differentiation of naive CD4^+^ T cells into Th1 cells and is induced by the activation of STAT1/4 downstream of IL-12 and IFN-γ signalling. Thus our data suggests that the induction of Tbet and differentiation of Th1 cells may be associated with CIS conversion, at least in a proportion of patients. Th1 cells were originally considered to be the key pathogenic cell type in MS, however more recently a role for Th17 cells has also emerged (Fletcher et al. 2010). Increased expression of both IFN-γ and IL-17, Th1 and Th17 cytokines respectively, have been associated with CIS and RRMS (Muls et al. 2012; Frisullo et al. 2008). We did not however observe any significant differences in the expression of RORC, the Th17 transcription factor. It is possible however, that our approach did not have sufficient sensitivity to detect possible alterations in RORC/Th17 cells, since this subset is present in blood at a much lower frequency than Th1 cells. Future studies in a larger cohort would benefit from the inclusion of additional analyses such as protein levels of T cell subset-specific transcription factors and cytokine production.

A number of other studies have attempted to identify correlates of CIS progression, with the aim of identifying patients at high risk of conversion to CDMS. A study by Corvol et al. used microarrays to study gene expression in naive CD4^+^ T cells from 37 CIS patients at time of diagnosis and after 1 year. On the basis of changes in gene expression at baseline, patients were divided into 4 groups, and 92 % of group 1 CIS patients converted to CDMS within 1 year (Corvol et al. 2008). Interestingly down-regulation of TOB1 and increased CD44 was characteristic of these rapidly progressing group 1 patients; however we were unable to replicate these findings in our small cohort since we observed no difference in TOB1 or CD44 expression between CIS patients that converted within 1 year compared with those who did not. Subsequently the same group also showed that TOB1^−/−^ mice exhibited exacerbated symptoms of experimental autoimmune encephalomyelitis (EAE), suggesting that TOB1 has an important role in constraining CNS autoimmunity (Schulze-Topphoff et al. 2013). The reasons for the discrepancy between our data and the studies described above are not clear, however it is possible that our cohort was not of sufficient size to identify differences in the expression of TOB1.

In conclusion this study suggests a possible role for increased expression of Tbet in CD4^+^ T cells as a biomarker for early conversion from CIS to CDMS and warrants further exploration in larger, longitudinal cohorts.

## References

[CR1] Baranzini SE (2014). The role of antiproliferative gene Tob1 in the immune system. Clin Exp Neuroimmunol.

[CR2] Corvol JC, Pelletier D, Henry RG, Caillier SJ, Wang J, Pappas D, Casazza S, Okuda DT, Hauser SL, Oksenberg JR, Baranzini SE (2008). Abrogation of T cell quiescence characterizes patients at high risk for multiple sclerosis after the initial neurological event. Proc Natl Acad Sci USA.

[CR3] Fletcher JM, Lalor SJ, Sweeney CM, Tubridy N, Mills KH (2010). T cells in multiple sclerosis and experimental autoimmune encephalomyelitis. Clin Exp Immunol.

[CR4] Frisullo G, Nociti V, Iorio R, Patanella AK, Marti A, Caggiula M, Mirabella M, Tonali PA, Batocchi AP (2008). IL17 and IFNgamma production by peripheral blood mononuclear cells from clinically isolated syndrome to secondary progressive multiple sclerosis. Cytokine.

[CR5] Miller D, Barkhof F, Montalban X, Thompson A, Filippi M (2005). Clinically isolated syndromes suggestive of multiple sclerosis, part I: natural history, pathogenesis, diagnosis, and prognosis. Lancet Neurol.

[CR6] Muls N, Jnaoui K, Dang HA, Wauters A, Van Snick J, Sindic CJ, van Pesch V (2012). Upregulation of IL-17, but not of IL-9, in circulating cells of CIS and relapsing MS patients. Impact of corticosteroid therapy on the cytokine network. J Neuroimmunol.

[CR7] Naor D, Sionov RV, Ish-Shalom D (1997). CD44: structure, function, and association with the malignant process. Adv Cancer Res.

[CR8] Nilsson P, Larsson EM, Maly-Sundgren P, Perfekt R, Sandberg-Wollheim M (2005). Predicting the outcome of optic neuritis: evaluation of risk factors after 30 years of follow-up. J Neurol.

[CR9] Optic Neuritis Study Group (2008). Multiple sclerosis risk after optic neuritis: final optic neuritis treatment trial follow-up. Arch Neurol.

[CR10] Schulze-Topphoff U, Casazza S, Varrin-Doyer M, Pekarek K, Sobel RA, Hauser SL, Oksenberg JR, Zamvil SS, Baranzini SE (2013). Tob1 plays a critical role in the activation of encephalitogenic T cells in CNS autoimmunity. J Exp Med.

[CR11] Shimizu Y, Van Seventer GA, Siraganian R, Wahl L, Shaw S (1989). Dual role of the CD44 molecule in T cell adhesion and activation. J Immunol.

[CR12] Tzachanis D, Freeman GJ, Hirano N, van Puijenbroek AA, Delfs MW, Berezovskaya A, Nadler LM, Boussiotis VA (2001). Tob is a negative regulator of activation that is expressed in anergic and quiescent T cells. Nat Immunol.

[CR13] Weber GF, Ashkar S, Glimcher MJ, Cantor H (1996). Receptor-ligand interaction between CD44 and osteopontin (Eta-1). Science.

